# Neuroanatomical correlates of reality monitoring in patients with schizophrenia and auditory hallucinations

**DOI:** 10.1192/j.eurpsy.2021.2234

**Published:** 2021-09-22

**Authors:** Mélanie Perret, Layla Lavallé, Frédéric Haesebaert, Marie-Françoise Suaud-Chagny, Jérôme Brunelin, Marine Mondino

**Affiliations:** 1 INSERM, U1028, CNRS, UMR5292, Lyon Neuroscience Research Center, Psychiatric Disorders: from Resistance to Response Team, Lyon F-69000, France; 2 University Lyon 1, Villeurbanne F-69000, France; 3 Centre Hospitalier Le Vinatier, Bron, France

**Keywords:** Paracingulate sulcus, reality monitoring, schizophrenia, source monitoring, structural neuroimaging

## Abstract

**Background:**

Reality-monitoring process enables to discriminate memories of internally generated information from memories of externally derived information. Studies have reported impaired reality-monitoring abilities in schizophrenia patients with auditory hallucinations (AHs), specifically with an exacerbated externalization bias, as well as alterations in neural activity within frontotemporoparietal areas. In healthy subjects, impaired reality-monitoring abilities have been associated with reduction of the paracingulate sulcus (PCS). The current study aimed to identify neuroanatomical correlates of reality monitoring in patients with schizophrenia.

**Methods:**

Thirty-five patients with schizophrenia and AHs underwent a reality-monitoring task and a 3D anatomical MRI scan at 1.5 T. PCS lengths were measured separately for each hemisphere, and whole-brain voxel-based morphometry analyses were performed using the Computational Anatomy Toolbox (version 12.6) to evaluate the gray-matter volume (GMV). Partial correlation analyses were used to investigate the relationship between reality-monitoring and neuroanatomical outcomes (PCS length and GMV), with age and intracranial volume as covariates.

**Results:**

The right PCS length was positively correlated with reality-monitoring accuracy (Spearman’s *ρ* = 0.431, *p* = 0.012) and negatively with the externalization bias (Spearman’s *ρ* = −0.379, *p* = 0.029). Reality-monitoring accuracy was positively correlated with GMV in the right angular gyrus, whereas externalization bias was negatively correlated with GMV in the left supramarginal gyrus/superior temporal gyrus, in the right lingual gyrus and in the bilateral inferior temporal/fusiform gyri (voxel-level *p* < 0.001 and cluster-level *p* < 0.05, FDR-corrected).

**Conclusions:**

Reduced reality-monitoring abilities were significantly associated with shorter right PCS and reduced GMV in temporal and parietal regions of the reality-monitoring network in schizophrenia patients with AHs.

## Introduction

Reality monitoring is a crucial cognitive process in the daily life to differentiate memories of thoughts and imagination from memories of externally derived information [1]. For instance, this process allows us to determine whether an event was generated by our imagination or if it really did occur.

A deficit in the reality-monitoring abilities has been repeatedly observed in patients with schizophrenia compared with healthy individuals (e.g., [2]; for recent review, see [3]). More specifically, several studies have pointed out that patients with schizophrenia and auditory hallucinations (AHs) were more likely to misattribute internally generated stimuli as being perceived from the environment than patients with schizophrenia without AHs and healthy individuals ([4–6]; for review, see [7,8]). This tendency to misattribute imagined events as being perceived is called an externalization bias and is assumed to partly underlie AHs. Indeed, a prominent cognitive model of AHs suggests that they might arise from a misattribution of internal mental events such as inner speech as being externally perceived [9,10].

The neural network underlying reality-monitoring process has been explored in both healthy individuals and patients with schizophrenia in the literature. The prefrontal cortex (PFC), and particularly its medial and anterior part, was found to be a key structure of this network (for review, see [3,11]). Interestingly, a functional neuroimaging study has reported that the externalization bias was correlated with a reduced activation in this specific brain region [12]. In patients with schizophrenia, deficits in the neural activity of the medial PFC have been observed during reality-monitoring performances [13,14]. The medial PFC is not the only brain region that may account for the reality-monitoring process. Indeed, the contribution of temporoparietal areas, and particularly their abnormal overactivation, into the experience of externalization bias has been supported by neuroimaging studies [15] as well as noninvasive brain stimulation studies [16].

Although neuroimaging studies have broadly investigated brain activity linked to reality-monitoring performances, less is known about the neuroanatomical correlates of reality monitoring. In recent years, the morphology of a specific structure of the medial PFC, the paracingulate sulcus (PCS), has been investigated. The PCS is a tertiary sulcus that lies in the medial wall of the PFC and runs dorsal and parallel to the cingulate sulcus in a rostro-caudal direction. The PCS presents a great morphological variability within the general population, in that it can be found in none, one, or both hemispheres [17], and its presence affects the morphometry [18,19] and the cytoarchitectonic organization of surrounding cortices [20,21]. The PCS was found to be associated with a wide array of executive and cognitive functions [22], including reality monitoring [23]. Namely, healthy individuals with bilaterally absent PCS showed significantly reduced reality-monitoring performances compared with individuals with present PCS in at least one hemisphere [23]. In schizophrenia patients, some studies showed that reduced PCS length was associated with AHs [24,25]. However, the relationship between the PCS length and reality-monitoring performances remains unclear in patients with schizophrenia and AHs. Particular anatomical features in the medial PFC and specific morphology of the PCS could underpin the relationship between brain activity within these areas and reality-monitoring process.

The present study aimed to identify whether reality-monitoring performances were linked to specific neuro-anatomical features, including PCS length and gray-matter volume (GMV), in hallucinating patients with schizophrenia. Therefore, we conducted a magnetic resonance imaging (MRI) study combining an investigation of reality-monitoring performances, a morphological analysis of the PCS, and a whole-brain voxel-based morphometry (VBM) analysis. We hypothesized that reality-monitoring deficits, and particularly the externalization bias, will be negatively correlated with the PCS length. These hypotheses were based on three lines of work presented above showing that: (a) the absence of PCS is associated with poor reality-monitoring performances [23], (b) shorter PCS length is associated with AHs [24], and (c) AHs are associated with a specific deficit in reality monitoring: the externalization bias [8]. In addition, we hypothesized that poorer reality-monitoring performances, including higher externalization bias, would be associated with lower GMV in the brain regions that were identified as functionally involved in reality monitoring [11] and in the externalization bias [12] (e.g., the medial PFC).

## Methods

### Participants

Thirty-five patients meeting the DSM-IV-TR criteria for schizophrenia were recruited from our clinical unit for treatment-resistant schizophrenia at Le Vinatier Hospital between 2009 and 2015. All participants were native French speakers and presented daily treatment-resistant AHs, defined as persistent daily AHs despite an antipsychotic treatment at an adequate dosage for more than 6 weeks. Patients’ diagnoses were assessed through a formal interview with a trained psychiatrist using the Mini-International Neuropsychiatric Interview [26]. Participants were assessed for the severity of their symptoms using the Positive and Negative Syndrome Scale (PANSS) [27]. Patients’ current antipsychotic medication classes (typical, atypical including clozapine, and combination of classes) were reported in Table 1. Written informed consent was obtained from all participants. All experiments were approved by a local ethic committee (CPP SUD EST VI, Clermont-Ferrand, France) and performed in compliance with relevant guidelines and regulations.

**Table 1.** Summary of demographic, clinical, and reality-monitoring measures and paracingulate sulcus (PCS) lengths of the 35 patients with schizophrenia and auditory hallucinations.

	*N*	Mean	SD	Range
Gender (men/women)	21/14			
Handedness (left/right)	4/31			
Age (years)		37.1	8.9	24–52
Education (years)		11.6	2.5	8–16
Duration of illness (years)		10.9	7.5	1–30
Antipsychotic medication				
Typical antipsychotics	14			
Atypical antipsychotics	31			
Including clozapine	12			
Combination of typical and atypical antipsychotics	10			
Positive and Negative Syndrome Scale^a^		71.6	14.7	45–110
Positive^a^		19.6	4.2	12–28
Negative^a^		19.1	6.5	9–40
General psychopathology^a^		32.9	7.7	18–47
Reality-monitoring task				
Reality-monitoring accuracy		0.73	0.18	0.42–1
Externalization bias		0.41	0.36	0–1
Item memory accuracy d’		2.11	0.74	0.30–3.48
Total intracranial volume (mm^3^)		1,496.3	150.1	1,229.0–1,921.9
Left PCS length (mm)		35.2	24.4	0–84.7
Right PCS length (mm)		21.6	20.8	0–78.5

*Abbreviation:* SD, standard deviation.

a *N* = 34 (one missing data).

### Reality-monitoring task

The task was divided in a presentation phase and a test phase, according to the task used and validated by Brunelin et al. [28]. Briefly, during the presentation phase, 16 words were presented one by one on a computer screen for 3 s, all preceded by an instruction also presented during 3 s. Instructions were “Imagine yourself hearing the following word” or “Listen to the following word.” During the test phase, performed immediately after the presentation phase, a 24-word list was presented including the 16 words previously presented (8 imagined and 8 listened) and 8 new words (distractors). Patients had to determine the source for each word (i.e., “Imagined,” “Heard,” or “New”). Before the task, patients performed a short practice trial to acquaint with requirements of the task and to ensure for their good comprehension.

Three main outcomes were computed according to previous studies [29,30]. (a) Reality-monitoring accuracy was calculated using the following formula:
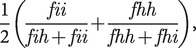
where 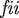 is the number of imagined words that were correctly recognized as imagined, 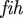 is the number of imagined words identified as being heard, 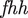 is the number of heard words correctly identified as heard, and 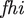 is the number of heard words identified as imagined. This measure of reality monitoring, also known as average conditional source identification measure [31], reflects the proportion of correct source judgments among the item correctly recognized as old. (b) The externalization bias was defined as the number of imagined words recognized as heard among all imagined words incorrectly judged (i.e., as new or heard). (c) Item memory accuracy was calculated as the standardized hit rate (*z*-score of hit rate, i.e., the proportion of old items identified as old) minus the standardized false alarm rate (*z*-score of false alarm rate, i.e., the proportion of new items identified as old). Before calculation, hit and false alarm rates were corrected to avoid the values of 0 and 1, as recommended by Snodgrass and Corwin [32]. This measure of item memory, also known as the Signal Detection Theory metrics’ *d’* [33], reflects the sensitivity to discriminate between old and new items.

### Magnetic resonance imaging acquisition

MRI acquisitions were performed on a 1.5-T Siemens Magneton scanner. A 3D anatomic T1-weighted sequence covering the whole brain volume was acquired with the following parameters: 176 transverse slices, TR = 1,970 ms, TE = 3.93 ms, field of view = 256 mm^2^, and voxel size = 1 mm^3^.

### Paracingulate sulcus measurements

**Figure 1.**
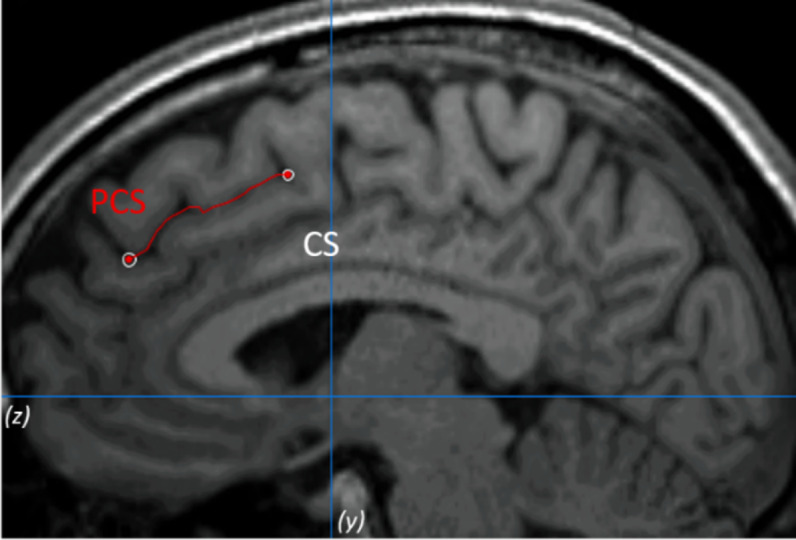
Example of the paracingulate sulcus (PCS) measurement on anatomical magnetic resonance imaging. The PCS is marked in red line and lies dorsal and parallel to the cingulate sulcus. Measurement was performed within the first quadrant (defined by *z* < 0 and *y* > 0) and on the fourth sagittal slice for both hemispheres.

The PCS was measured following the measurement protocol described by Garrison et al. [25] (see Figure 1 as an example). To validate the procedure, inter- and intrarater reliabilities were calculated. See the Supplementary Material for more details.

### Voxel-based morphometry analysis

All images were preprocessed and analyzed with the Computational Anatomy Toolbox (CAT, version 12.6; http://www.neuro.uni-jena.de/cat/) implemented in Statistical Parametric Mapping (SPM12) (Welcome Trust Center for NeuroImaging, London, UK; http://www.fil.ion.ucl.ac.uk.gate2.inist.fr/spm/software/spm12/) using MATLAB (R2018a, MathWorks, Inc., Massachusetts, USA). Both processing and analysis were performed following the standard protocol (http://www.neuro.uni-jena.de/cat12/CAT12-Manual.pdf) with default settings, unless otherwise indicated. This method has been previously validated and provides a great compromise between good quality and speed of processing [34]. Prior to preprocessing, each image was visually inspected for artifacts. Then, T1 images were corrected for bias field inhomogeneities, segmented into gray matter, white matter, and cerebrospinal fluid, spatially normalized into a standard Montreal Neurological Institute (MNI) space using the DARTEL algorithm, and modulated to allow comparison of the absolute amount of tissue. A second quality control for intersubject homogeneity and overall image quality was achieved using the automated quality check protocol of the CAT12 toolbox. After quality check, the total intracranial volume of each subject was estimated to be used as covariate on the second-level analyses to take into account intersubjects brain size variations. Finally, images were smoothed using an 8-mm Full Width-Half Max (FWHM) kernel.

### Statistical analyses

Statistical analyses were conducted using R software (version 3.5.2)[35]. Normality of the data was tested using the Shapiro–Wilk test. Partial Spearman’s rank correlations were calculated to assess the relationship between PCS lengths (separately for each hemisphere) and outcomes of the reality-monitoring task (reality-monitoring accuracy, externalization bias, and item memory), with total intracranial volume and age as confounding variables. For all analyses, a significance level of *p* < 0.05 was employed. As exploratory analyses, we investigated whether PCS lengths were also related to total positive symptoms, by computing partial Spearman’s rank correlations between PCS lengths and total PANSS positive scores, with total intracranial volume and age as confounding variables.

VBM statistical analyses were performed with the CAT12 toolbox (version 12.6). A multiple linear regression model was used to test for voxel-wise correlations between GMV and reality-monitoring outcomes. Total intracranial volume and age were used as confounding covariates in these analyses. A 0.1 absolute masking threshold was applied to avoid artifact on the gray/white matter limit. For all voxel-based analyses, we thresholded statistical maps with an uncorrected *p* < 0.001 at voxel level and with an false discovery rate (FDR)-corrected *p* < 0.05 at the cluster level. Significant clusters were labeled using the Anatomical Automatic Labelling in SPM.

## Results

Patients’ demographic and clinical characteristics, as well as reality-monitoring outcomes, total intracranial volumes, and PCS lengths for each hemisphere, are presented in Table 1. Details on patients’ scores at each individual item of the PANSS positive subscale are provided in the Supplementary Material.

### Reality monitoring and PCS length

**Figure 2.**
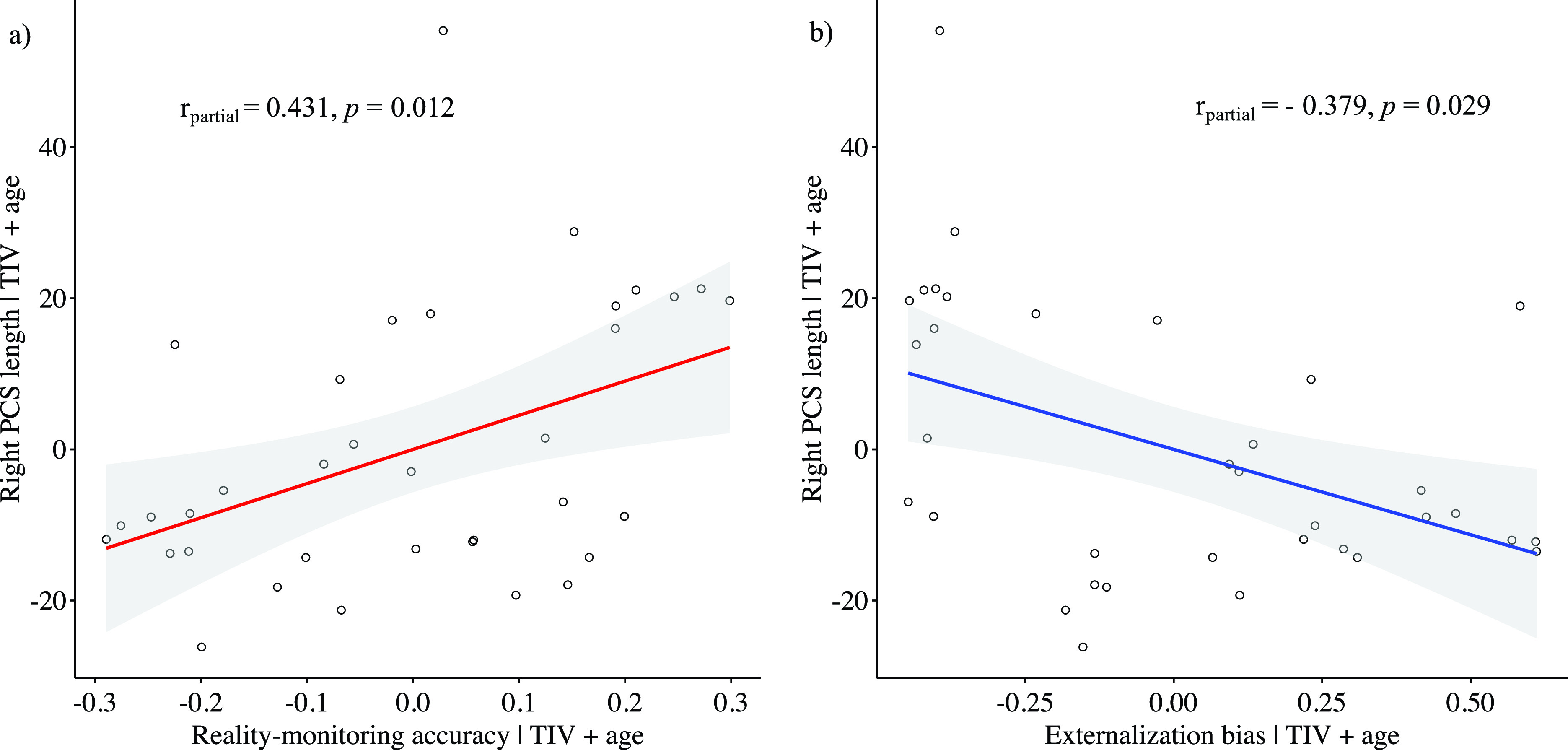
Scatter plots showing the partial correlations between the paracingulate sulcus length in the right hemisphere and (A) reality-monitoring accuracy and (B) the externalization bias, controlling for total intracranial volume and age (*n* = 35).

While controlling for age and total intracranial volume, the PCS length was positively correlated with reality-monitoring accuracy in the right hemisphere (Spearman’s partial *ρ* = 0.431, *p* = 0.012; Figure 2A) but not in the left hemisphere (Spearman’s partial *ρ* = 0.052, *p* = 0.773). There was a significant negative correlation between the length of the right PCS and the externalization bias (Spearman’s partial *ρ* = −0.379, *p* = 0.029; Figure 2B), but no significant correlation was found for the left PCS length and the externalization bias (Spearman’s partial *ρ* = 0.171, *p* = 0.340). No significant correlations were found between PCS lengths and item memory (for the right PCS: Spearman’s partial *ρ* = 0.137, *p* = 0.448; for the left PCS: Spearman’s partial *ρ* = −0.003, *p* = 0.988).

Exploratory analyses revealed no significant correlations between PCS lengths and total PANSS positive scores (for the right PCS: Spearman’s partial *ρ* = −0.021, *p* = 0.908; for the left PCS: Spearman’s partial *ρ* = −0.108, *p* = 0.555).

### Reality monitoring and GMV

**Figure 3.**
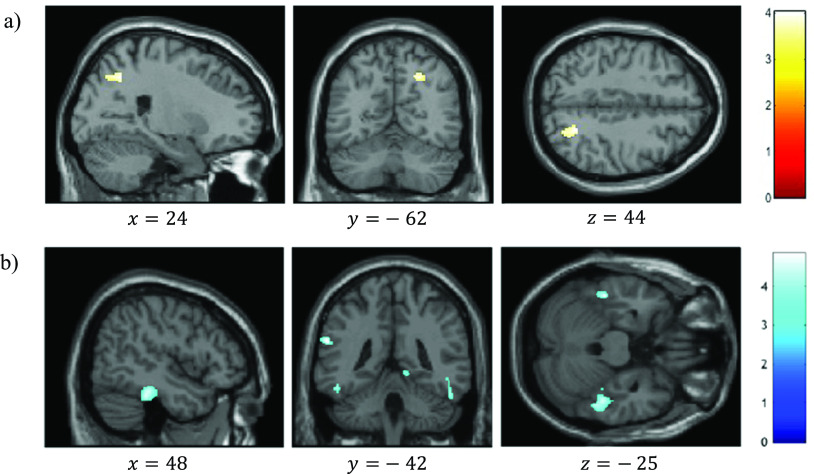
Voxel-based morphometry analysis of correlation between gray-matter volumes and reality-monitoring performances, corrected for total intracranial volume and age (*n* = 35). Results are reported using *z*-values presented in the SPM canonical single subject template with MNI coordinates of the section. Regions that survived a statistical threshold of *p* < 0.001 at the peak-level (FDR-corrected *p* < 0.05 at the cluster level) are shown in (A) for positive correlation with reality-monitoring accuracy and (B) for negative correlation with externalization bias.

VBM analysis revealed a significant positive correlation between reality-monitoring accuracy and GMV in the right angular gyrus (peak MNI coordinates [23 −59 44], *t* = 4.03, *p* < 0.001; see Table 2 and Figure 3A).

**Table 2.** Clusters showing significant correlations between gray-matter volume and reality-monitoring measures (*n* = 35).

Reality-monitoring scores	Correlation	Anatomical regions	Cluster size	Coordinates (mm)			*t*-value
				*x*	*y*	*z*	
Reality-monitoring accuracy	Positive	Right angular gyrus	175	23	−59	44	4.03
Externalization bias	Negative	Left supramarginal gyrus/superior temporal gyrus	88	−60	−42	24	4.83
		Right lingual gyrus	236	15	−51	−3	4.71
		Left inferior temporal gyrus/fusiform gyrus	189	−46	−33	−26	4.60
		Right inferior temporal gyrus/fusiform gyrus	540	48	−33	−24	4.48
Item memory	Negative	Right superior frontal gyrus	185	26	45	22	4.57

*Note:* Statistical threshold of *p* < 0.001 at the peak-level and FDR-corrected *p* < 0.05 at the cluster level.

The analysis also revealed a significant negative correlation between the externalization bias and GMV in a cluster encompassing the left supramarginal gyrus and the left superior temporal gyrus ([−60 −42 24], *t* = 4.83, *p* < 0.001), in the right lingual gyrus ([15 −51 −3], *t* = 4.71, *p* < 0.001), and both in the left and the right inferior temporal gyrus and fusiform gyrus (respectively, [−46 −33 −26], *t* = 4.60, *p* < 0.001, and [48 −33 −24], *t* = 4.48, *p* < 0.001; Table 2 and Figure 3B).

Additional VBM analyses revealed a significant negative correlation between item memory and GMV in the right superior frontal gyrus (peak MNI coordinates [26 45 22], *t* = 4.57, *p* < 0.001; Table 2).

## Discussion

The present study sought to identify the neuroanatomical correlates of reality monitoring in a sample of schizophrenia patients with AHs. We reported two main findings: (a) the right hemisphere PCS length was positively correlated with reality-monitoring accuracy and negatively correlated with the externalization bias, that is, the misattribution of imagined words to an external source, and (b) the reality-monitoring accuracy was positively correlated with the GMV in the right angular gyrus, whereas the externalization bias was negatively correlated with the GMV in a set of temporal and parietal areas.

We demonstrated a significant correlation between the reality-monitoring abilities of hallucinating patients with schizophrenia and the length of the PCS in the right hemisphere: the shorter the PCS, the poorer the reality-monitoring accuracy and the greater the externalization bias. On the one hand, these results are highly coherent with those found in healthy subjects associating the absence of PCS with worse overall reality-monitoring accuracy [23]. On the other hand, the region containing the PCS has been associated with both AHs and reality-monitoring abilities [23–25]. Moreover, a recent study has demonstrated that this region causally supports reality monitoring. In healthy subjects, active real-time fMRI neurofeedback training of the paracingulate cortex has been reported to improve the reality-monitoring accuracy for imagined items as well as the functional activity of the paracingulate cortex [36]. If the relationship between the PCS morphology and the functional role of the paracingulate cortex remains unclear, taken together these findings suggest that the PCS morphology may be the structural basis for the causal role of the paracingulate cortex in reality-monitoring abilities and hallucinations. Indeed, the PCS morphology is known to influence the topography of the medial PFC [21] and to generate a great interindividual variability on the location of the neural activity evoked in the medial PFC during a given cognitive task in healthy subjects [37]. Future fMRI studies should consider this morphological variability when reporting differences in brain activity in the medial PFC during reality-monitoring paradigms. The differences in medial PFC activity observed at the group level during a reality-monitoring task could reflect a different location of the neural activity due to intersubject differences in the PCS morphology. Taking into account this neuroanatomical feature when studying functional patterns of reality monitoring would provide more reliable evidence of a deficit in populations experiencing AHs.

It is noteworthy that the PCS is one of the latest sulci to develop in utero, appearing at the 36th week of ontogeny and maturing to the perinatal period for human [22,38]. This sulcus is thus exposed to environmental factors able to interfere with its development. The reality-monitoring impairment found to be correlated with the PCS length may thus result from defective neurodevelopmental mechanisms. In this line, abnormal reality-monitoring performances have also been observed before the onset of frank psychotic episode in individuals at risk for schizophrenia within the continuum of psychosis [39]. A deepen investigation of the sulcal ontogeny, and even more of the developmental factors that may influence the PCS morphometry could improve the understanding of its relationship with reality-monitoring deficits.

Consistent with the right lateralization of our findings, a recent study found a reduction of the PCS length only in the right hemisphere of both psychotic and nonclinical voice hearers [40], suggesting the right PCS length reduction to be a specific marker of AHs whatever the clinical condition. By contrast, some studies identified bilateral PCS reductions in schizophrenia patients with AHs as compared with schizophrenia patients without AHs, nonclinical subjects with AHs, and healthy controls [24,25], and some others found specific left PCS reduction in schizophrenia patients with AHs as compared with those without AHs, and healthy controls [41]. Further studies are thus needed to clarify if the length of the right PCS may be considered as a specific neuroanatomical marker of AHs or if the bilateral PCS is only reduced in schizophrenia patients with AHs.

Surprisingly, reality-monitoring performances did not correlate with GMV in medial frontal areas. Yet, the functional capacity of the medial PFC has been largely involved in the reality-monitoring process in both patients with schizophrenia and healthy individuals [13,42], and reduced GMV has been observed in these brain areas in patients with schizophrenia [43]. In addition, the presence/absence of PCS has been associated with GMV in the surrounding frontal regions, and these volumetric changes were related to reality-monitoring performances [23]. Further studies are now needed to investigate a potential relationship between the PCS variability and the surrounding prefrontal volume and its implication on the prefrontal functional capacity during reality monitoring.

As we hypothesized, most of the regions for which the GMV correlated with reality-monitoring performances correspond to the temporoparietal areas previously identified by functional imaging during reality-monitoring tasks. We found several brain structures whose GMVs negatively correlate with the externalization bias, indicating that schizophrenia patients with AHs with reduced GMV in these structures are more likely to misattribute internally generated information to an external source.

First, we observed a negative correlation between the externalization bias and a cluster encompassing the left supramarginal gyrus and the left superior temporal gyrus, which is considered as a part of Wernicke’s area (BA 40) involved in auditory and speech processing. Disruption to this system would induce an inadequate treatment of the verbal items presented in reality-monitoring tasks and participate to patients’ misattributions of source. In addition, a recent meta-analysis on motor agency specifically highlighted the left BA 40 as an integral part of the body-ownership network [44]. This cluster can thus be considered as an element of both verbal and nonverbal self-production recognition, suggesting its modality-general implication in reality-monitoring processes. Consistently, the GMV and activity of this temporoparietal region have also been associated with AHs in schizophrenia patients [45–47]. The causal implication of temporoparietal regions in reality monitoring has finally been demonstrated by noninvasive stimulation over this region that modulated the externalization bias in both healthy subjects and schizophrenia patients and alleviated AHs in schizophrenia patients [16, 48–50].

The VBM analysis also revealed negative correlations between the externalization bias and gray matter in several posteroinferior temporal regions. Considered as associative visual areas, these structures have mainly been associated with visual processing and visual hallucinations [47,51,52]. For now, the implications of the correlation between their GMV and externalization bias in our semantic task are unclear, and future studies should clarify the relationship between reduced GMV in these areas and the incorrect source attributions observed in schizophrenia patients with AHs. However, a substantial body of functional studies has already reported an activation of the right lingual gyrus during Theory-of-Mind tasks, involving among other things to make the distinction between internal and external space [53]. On its side, the left inferior temporal gyrus has been shown to specifically activate in the reality-monitoring contrast “correct attributions” versus “misattributions” in healthy participants [54].

We identified a significant positive correlation between the reality-monitoring accuracy and the GMV of the right angular gyrus. This result replicates in a population of schizophrenia patients with AHs the results reported by Buda et al. [23] in a sample of healthy subjects. The right angular gyrus is engaged in a wide range of tasks reflecting our ability to discriminate the internal from external environment, such as Theory-of-Mind or agency attribution tasks [55,56]. Moreover, several case reports described its causal involvement in out-of-body experiences, a phenomenon referring to an autoscopic experience during which the subject perceive the world from an out-of-body position [57,58]. In this way, our findings contribute to define the right angular gyrus as a pivotal neural locus for the distinction between the self and the external world. Its increased GMV may underlie its overactivity and in turn sustain decreased reality-monitoring performances in schizophrenia patients with AHs.

In addition to the sample size that could be considered as limited for correlation analyses (estimated post hoc power of 0.75), the main limitation of this study is the lack of comparison groups. Additional groups of healthy participants, healthy voice hearers, and patients with schizophrenia without AHs would had allowed us to determine if the structural correlates of reality monitoring are specific to schizophrenia or if they could be expanded to the global population. However, despite this limitation, our study has the advantage of investigating reality monitoring in a homogeneous sample of patients with severe daily treatment-resistant AHs, as compared with mixed samples of patients with heterogenous symptoms that are usually enrolled in the literature. The particularity of our patient sample in terms of treatment resistance and severity of AHs might also contribute to the differences in the right PCS length observed between our study and other studies including patients with AHs [24,25,41]. Second, the question of the specificity of findings reported in the current study remains open. VBM findings suggested that reality-monitoring performances and item memory were linked to GMV changes in different brain regions. In addition, the PCS length seems to be specifically linked to reality-monitoring performances, that is, to reality-monitoring accuracy and externalization bias, but not to item memory or total positive symptoms. However, further investigations might assess whether reality monitoring might be related to other sulci. Third, one could question how the PCS, which can be considered as a static brain structure, could be related to a dynamic process such as reality monitoring. Although the PCS is expected to remain stable after its maturation during perinatal period, some PCS length changes over time have been described in a longitudinal study with adolescent onset psychosis [59]. Nevertheless, the observed correlation of reality-monitoring outcomes and PCS lengths does not necessarily imply that the PCS length is the only anatomical substrate for reality-monitoring deficits (and the emergence of AHs). Rather, we could hypothesize a two-hit process with a reality-monitoring deficit that predates the emergence of AHs, since reality-monitoring deficits are also reported in people with an at-risk mental state for psychosis and unaffected relatives of patients with schizophrenia [39], and which might be linked to the PCS length, and a second phase of aggravation of reality-monitoring deficits, together with other neuroanatomical features, such as GMV alterations.

In summary, this study demonstrated that reality-monitoring performances correlated with both the PCS morphology and the GMV in crucial brain regions engaged in the reality-monitoring neural network in patients with schizophrenia. If the exact relationship between the structural evidence that we have highlighted and their functional implications remains little known, these correlations propose some anatomical substrates for the observed reality-monitoring errors in schizophrenia patients with AHs. Such associations would lead future studies to clarify the relationship between the PCS and GMV variability and reality-monitoring abilities. Finally, further research work should investigate if similar structural features would be associated with AHs in nonclinical hallucinating individuals or if they specifically characterize AHs in schizophrenia.

## Data Availability

The data that support the findings of this study are available from the corresponding author, M.M., upon reasonable request.

## Supplementary Materials

To view supplementary material for this article, please visit http://dx.doi.org/10.1192/j.eurpsy.2021.2234. click here to view supplementary material
