# Supplementary material for: Neuroanatomical correlates of reality monitoring in patients with schizophrenia and auditory hallucinations
Source: Eur Psychiatry. 2021 Sep 22;64(1):e58. doi: 10.1192/j.eurpsy.2021.2234 (PMC8516745; doi:10.1192/j.eurpsy.2021.2234)
Supplement: Supplementary file 1 [file S0924933821022343sup001.docx]

**Supplementary Material**

**Methods**

Paracingulate Sulcus measurements

The paracingulate sulcus (PCS) was measured using Mango brain visualization software (version 4.0.1; <http://ric.uthscsa.edu/mango>) following the measurement protocol described by Garrison et al. [1]. T1 images were individually straightened in an orthonormal landmark and the anterior and posterior commissures (AC and PC) were aligned on the same horizontal plane. In the sagittal plane, the origin of the landmark was reset to the AC location and PCS were measured within the first quadrant delineated by y > 0 and z < 0 (Figure 1). The first step of sulcus identification was to locate the cingulate sulcus on a sagittal slice. It is the first major sulcus running in an anterior-posterior direction, dorsal to the corpus callosum and visible on 5 sagittal slices at least. PCS is defined as a salient sulcus running parallel, horizontal and dorsal to the cingulate sulcus and present on 3 sagittal slices at least. Using ‘trace line’ function, PCS was measured on a sagittal slice, 4 mm to the left or right of the medial line (x= ± 4), from the point at which the sulcus ran in a posterior direction to its end point which is possibly out of the first quadrant. Segments of discontinuous PCS were measured only if they started within the first quadrant and were separated by less than 20mm in length.

Intra- and inter-rater reliabilities for the PCS measurement

To validate the PCS measurement procedure, the rater who completed all PCS measurements (MP) repeated the PCS measurement for 16 randomly chosen subjects (32 hemispheres). In addition, each PCS of the 35 participants was independently measured by a second rater. Both raters were blind to patients details at all times during measurements. Intra- and inter-rater reliabilities assessed using intraclass correlation coefficients were respectively 0.908 (IC 95% [0.822; 0.954]) and 0.819 (IC 95% [0.724; 0.883]). Intraclass correlation coefficients values between 0.75 and 0.9 indicate a good reliability and values greater than 0.9 indicate an excellent reliability [2].

[1] Garrison JR, Fernyhough C, McCarthy-Jones S, Haggard M, Australian Schizophrenia Research Bank, Simons JS. Paracingulate sulcus morphology is associated with hallucinations in the human brain. Nat Commun 2015;6:8956. https://doi.org/10.1038/ncomms9956.

[2] Koo TK, Li MY. A Guideline of Selecting and Reporting Intraclass Correlation Coefficients for Reliability Research. J Chiropr Med 2016;15:155–63. https://doi.org/10.1016/j.jcm.2016.02.012.

**Supplementary Table 1.** Details on patients’ scores at each individual item of the PANSS-positive subscale *

|  | **Mean** | **SD** | **Range** |
| --- | --- | --- | --- |
| PANSS-P1: Delusions | 3.97 | 1.27 | 1 - 6 |
| PANSS-P2: Conceptual disorganization | 2.50 | 1.46 | 1 - 6 |
| PANSS-P3: Hallucinatory behavior | 5.85 | 0.86 | 4 - 7 |
| PANSS-P4: Excitement | 1.32 | 0.88 | 1 - 5 |
| PANSS-P5: Grandiosity | 1.62 | 1.21 | 1 - 6 |
| PANSS-P6: Suspiciousness / persecution | 3.15 | 1.11 | 1 - 5 |
| PANSS-P7: Hostility | 1.18 | 0.63 | 1 - 4 |

PANSS = Positive And Negative Syndrome Scale; SD = standard deviation. *PANSS items are rated on a 7-point scale (1=absent, 2=minimal, 3=mild, 4=moderate, 5=moderate severe, 6=severe, and 7=extreme).*

*** N = 34 (one missing data)


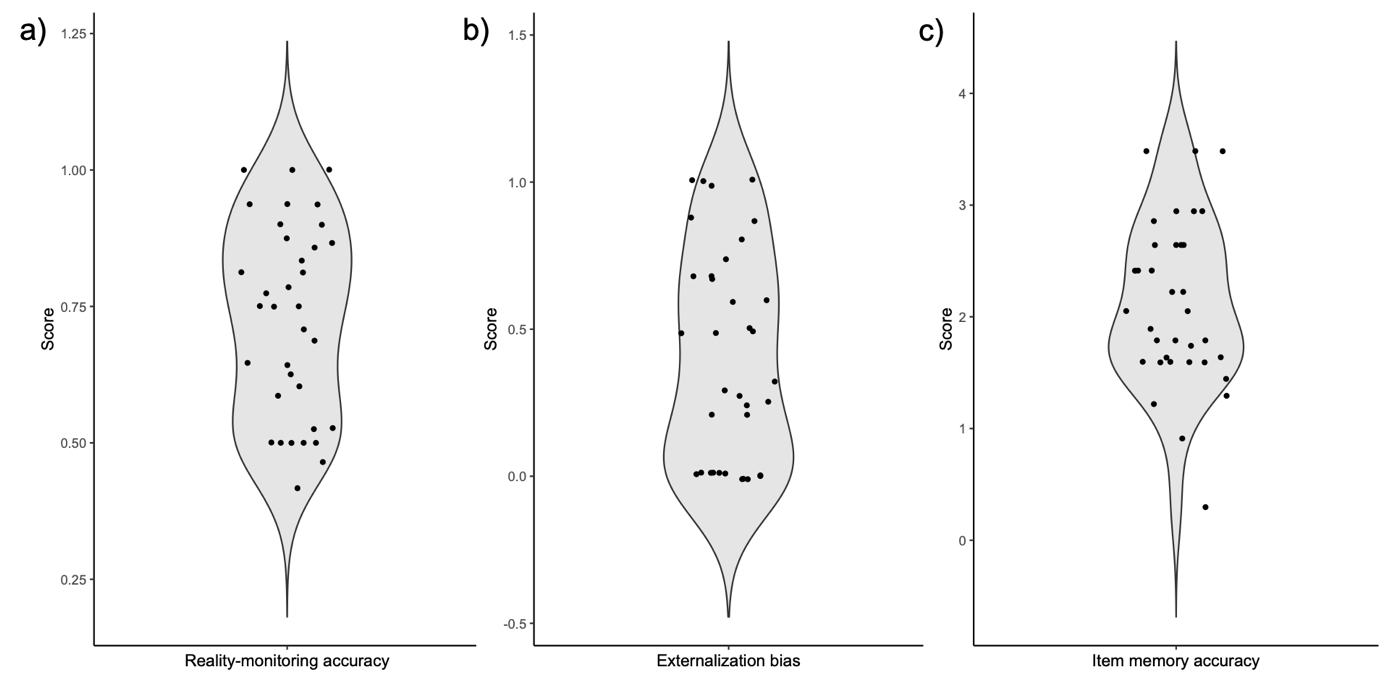
**Supplementary Figure 1.** Violin plots of the distribution of outcomes at the reality-monitoring task in the sample of 35 patients with schizophrenia and auditory hallucinations. Outcomes include: a) reality-monitoring accuracy calculated as the proportion of correct source recognition among the items correctly recognized as old, averaged across both sources (imagine and heard); b) externalization bias, defined as the number of imagined items recognized as heard among all imagined items incorrectly judged (*i.e.,* judged as new or heard); and c) item memory accuracy, calculated by subtracting the standardized false alarm rates (*i.e.,* the proportion of new items identified as old) from the standardized hit rates (*i.e.,* the proportion of old items identified as old).

**Supplementary Figure 2.** Violin plots of the distribution of paracingulate sulcus (PCS) length for each hemisphere in the sample of 35 patients with schizophrenia and auditory hallucinations. PCS lengths are expressed in mm.
